# Regulatory sRNAs in Cyanobacteria

**DOI:** 10.3389/fmicb.2018.02399

**Published:** 2018-10-19

**Authors:** Jinlu Hu, Qiang Wang

**Affiliations:** ^1^School of Life Sciences, Northwestern Polytechnical University, Xi’an, China; ^2^Key Laboratory of Algal Biology, Institute of Hydrobiology, The Chinese Academy of Sciences, Wuhan, China; ^3^University of the Chinese Academy of Sciences, Beijing, China

**Keywords:** small RNA, cyanobacteria, sRNA identification, regulatory functions, physiological roles

## Abstract

As the transcriptional and post-transcriptional regulators of gene expression, small RNAs (sRNAs) play important roles in every domain of life in organisms. It has been discovered gradually that bacteria possess multiple means of gene regulation using RNAs. They have been continuously used as model organisms for photosynthesis, metabolism, biotechnology, evolution, and nitrogen fixation for many decades. Cyanobacteria, one of the most ancient life forms, constitute all kinds of photoautotrophic bacteria and exist in almost any environment on this planet. It is believed that a complex RNA-based regulatory mechanism functions in cyanobacteria to help them adapt to changes and stresses in diverse environments. Although lagging far behind other model microorganisms, such as yeast and *Escherichia coli*, more and more non-coding regulatory sRNAs have been recognized in cyanobacteria during the past decades. In this article, by focusing on cyanobacterial sRNAs, the approaches for detection and targeting of sRNAs will be summarized, four major mechanisms and regulatory functions will be generalized, eight types of *cis*-encoded sRNA and four types of *trans*-encoded sRNAs will be reviewed in detail, and their possible physiological functions will be further discussed.

## Introduction

RNAs from cells that do not display messenger RNAs (mRNAs), ribosomal RNAs (rRNAs), or transfer RNAs (tRNAs) functions include a wide class of molecules, usually denominated as small RNAs (sRNAs) or non-coding RNAs (ncRNAs) (Storz et al., 2004; Waters and Storz, 2009). As the transcriptional and post-transcriptional regulators of gene expression, sRNAs play important roles in every domain of life in organisms (Raghavan et al., 2011). Different from protein-coding regions specified by genetic codes, sRNAs have no clear-cut signatures that represent their boundaries as regulatory RNAs or even their occurrence in a genome. They play key roles in various basic processes in organisms, including structural maintenance of chromosome (Storz, 2002; Volpe et al., 2002), regulation of stability and translation of mRNAs (Storz et al., 2004), translocation and stability of proteins (Huttenhofer et al., 2005; Hüttenhofer and Vogel, 2006), metabolic reactions (Park et al., 2010), stress responses (Romby et al., 2006), and pathogenesis (Lee and Groisman, 2010).

In prokaryotes, the length of sRNAs usually ranges from 50 to 500 nucleotides (nt). The sRNAs were known in prokaryotes as regulators for years before the first microRNA (miRNA) and short interfering RNA (siRNA) were found in eukaryotes. It was reported that a ∼108 nt RNA I blocked ColE1 plasmid replication by base pairing with the RNA, which was cleaved to produce the replication primer (Stougaard et al., 1981; Tomizawa et al., 1981). Shortly after that, a ∼70 nt RNA transcribed from the pOUT promoter of Tn10 transposon was discovered preventing translation of the transposase mRNA and repressing transposition (Simons and Kleckner, 1983). Subsequently, the 174 nt MicF RNA was identified as the first chromosomally encoded sRNA in *Escherichia coli* (*E. coli*) and plays a role in inhibiting translation of the mRNA encoding OmpF, the major outer membrane porin (Mizuno et al., 1984). These sRNAs were identified by multicopy phenotypes, by gel analysis due to their abundance, or by serendipity (Wassarman et al., 1999; Waters and Storz, 2009). During the past decade, the importance of this amazing group of RNAs has been widely recognized in various organisms (Mattick, 2001; Huttenhofer et al., 2002; Storz, 2002; Huttenhofer et al., 2005; Raghavan et al., 2011). Due to recent technical advancements, e.g., deep sequencing, computational searches, and tilling microarrays with full-genome coverage (Weinberg et al., 2007; Landt et al., 2008; Livny et al., 2008; Sittka et al., 2008), many new candidate sRNAs have now been predicted in *E. coli* and other bacteria. In *E. coli*, systematic computational analyses have predicted the existence of hundreds of sRNAs in the past few decades (Livny and Waldor, 2007); however, only about 80 sRNAs have been experimentally validated (Waters and Storz, 2009). Above all, the function of many sRNAs remains unknown.

Cyanobacteria constitute a wide variety of photoautotrophic bacteria that undergo oxygenic photosynthesis and grow under extremely diverse environmental conditions on Earth, such as oceans, freshwater, rocks surface, desert soils, or even polar regions. The existence of cyanobacteria can be traced back possibly up to 3–5 billion years (Schopf, 1993), and they have long been recognized as important model organisms for research in aspects such as photosynthesis (Pisciotta et al., 2010; Chen et al., 2015), metabolism (Tamagnini et al., 2002), stress responses (Wang et al., 2008; Ma et al., 2017), biotechnology (Abed et al., 2009; Zhan and Wang, 2018), evolution (Flores and Herrero, 2008), and nitrogen fixation (Goebel et al., 2010). Particularly, cyanobacteria are a group of unicellular aquatic prokaryotes that possess certain properties such as fast growth, short life cycle, and spontaneously transformability, which have entitled them to be one of simple experimental systems and the most promising feedstock for bioenergy generation (Quintana et al., 2011; Giordano and Wang, 2018).

The majority of data on bacterial sRNAs originated from enterobacteria analysis. To date, in cyanobacteria, identified or functionally characterized sRNAs have only been reported in a few studies. In our previous works, by deep RNA-sequencing (RNA-seq), a total number of 5261 putative sRNAs in *Synechocystis* sp. PCC 6803 were revealed from the genome and its four megaplasmids, providing a comprehensive overview of sRNAs in the model organism (Xu et al., 2014; Hu et al., 2017). It is very likely that cyanobacteria, present in almost any environment with a long evolutionary history, have evolved a complex RNA-based regulatory network to respond to environmental changes and stress, analogously to enterobacteria. Here, we will review the research works on cyanobacterial sRNAs, including approaches for sRNAs detecting and targeting, their mechanisms of action and regulatory functions, and their possible physiological functions.

## Identification of sRNAs and Their Targets in Cyanobacteria

Due to divergence in sequences, structures, and functions, cyanobacterial sRNAs have no common identifiers. Nevertheless, biocomputational prediction, tiling microarrays, and pyrosequencing revealed that many candidate sRNAs have been existing in some cyanobacterial model organisms for the past several decades. The combination of computational and experimental methods used to identify sRNAs can help us understand sRNAs better and is likely to provide the complete sRNAs definition soon.

### Biocomputational Prediction

The most important aspect of sRNA research includes designing the methods used for the prediction of sRNA and their targets. These investigations have been carried out in-depth in the last few years. The standard procedures used to predict bacterial sRNAs and their targets include these three steps: 1. identifying conserved sequences and/or structure features in intergenic regions, 2. clustering and comparing of pairwise or multiple alignments, and 3. scoring based on structural features of predicted RNAs, which were reviewed in detail by Backofen and Hess (2010).

A mass of sRNAs has been predicted based on comparative genomics analysis and non-comparatives screenings (Backofen and Hess, 2010; Kopf et al., 2014). Meanwhile, as a key bottleneck, the targets identification of these sRNAs is an immediate requirement for further progress in this field. Many developed target-prediction tools and related software for bacteria may be worth learning (**Table 1**). Some examples include TargetRNA (Tjaden et al., 2006; Tjaden, 2008), sequence-based scoring combined with stacking (Mandin et al., 2007), RNAplex (Tafer and Hofacker, 2008), sRNATarget (Zhao et al., 2008; Cao et al., 2009), RNAup (Muckstein et al., 2006), RNAhybrid (Rehmsmeier et al., 2004), GLASSgo (Lott et al., 2018), especially CopraRNA (Wright et al., 2014), and IntaRNA (Busch et al., 2008). Among those, CopraRNA is the latest asset of the Freiburg RNA tools Web server, which incorporates and extends the functions of IntaRNA to better predict targets and interaction domains of bacterial sRNA. The GLASSgo allows discovery of the homologous sRNA sequences, which is often the first step in the functional characterization and targets prediction of a sRNA. All of the earlier mentioned tools can be used to predict targets of cyanobacterial sRNA, if used reasonably. There is a representative report that possible sRNAs target genes were predicted using the IntaRNA tool, and then functional categorization analysis using gene ontology (GO) assignment programs and functional annotation tool DAVID (Dennis et al., 2003; Huang et al., 2008) can be used, evidencing that sRNAs are involved in various metabolic pathways (Xu et al., 2014).

**Table 1:** Summary of sRNA targets prediction tools mentioned in the text.

Tools	Algorithm	Advantage	Limitation	Source
TargetRNA	Target prediction based on sequence	1. Simplicity 2. Easy calculating of the significance of the found matches	Neglecting some factors that may contribute to RNA-RNA interaction, such as RNA secondary structure or the role of Hfq	Tjaden et al., 2006; Tjaden, 2008
Sequence-based scoring combined with stacking	Target prediction based on sequence	1. Simplicity 2. Easy calculating of significance of the found matches	Neglecting some factors that may contribute to RNA-RNA interaction, such as RNA secondary structure or the role of Hfq	Mandin et al., 2007
RNAplex	Thermodynamic scoring of mRNA-sRNA mixed duplexes	1. Providing a much more realistic model of RNA-RNA interaction, as compared to approaches based on sequence complementarity 2. Very fast 3. Easy calculating of significance of the found hits	Neglecting intra-molecular base pairs	Tafer and Hofacker, 2008
sRNATarget	Thermodynamic scoring of mRNA-sRNA mixed duplexes	1. Providing a much more realistic model of RNA-RNA interaction, as compared to approaches based on sequence complementarity 2. Very fast 3. Easily calculating the significance of the found hits	Neglecting intra-molecular base pairs	Zhao et al., 2008; Cao et al., 2009
RNAhybrid	Thermodynamic scoring of mRNA-sRNA mixed duplexes	1. Providing a much more realistic model of RNA-RNA interaction, as compared to approaches based on sequence complementarity 2. Very fast 3. Easy calculating of significance of the found hits	Neglecting intra-molecular base pairs	Rehmsmeier et al., 2004
RNAup	Handling accessibility in mRNA-sRNA interactions	Easily combined with faster methods for assessing RNA–RNA interactions, such as RNAhybrid and RNAplex	By itself not fast enough for genome-wide predictions of microRNA or siRNA targets	Muckstein et al., 2006
IntaRNA	Handling accessibility in mRNA-sRNA interactions	Including extensive postprocessing methods such as functional enrichment analysis and visualization of interacting regions	Predicting interactions in single organisms	Busch et al., 2008
CopraRNA	Handling accessibility in mRNA-sRNA interactions	Incorporating and extending the functionality of IntaRNA to better predict targets and interaction domains of sRNA		Wright et al., 2014
GLASSgo	Combines an iterative BLAST strategy with pairwise identity filtering and a graph-based clustering method	1. Very fast and high specificity 2. Easy-to-use		Lott et al., 2018

However, bioinformatic analysis alone is still full of high false positive rates. There are three main reasons for high false positive rates in cyanobacteria: (1) most target genes are at different genomic loci relative to their sRNAs; (2) although some sRNAs have single targets, others could regulate a multitude of gene expression; and (3) targets predictions of protein-modifying sRNAs also can be a challenge depending on the nature and understanding of the target characteristics. It is significantly necessary to identify more sRNAs targets of cyanobacteria by combining computational and experimental approaches in future. Georg et al. (2014) proved the interaction between the ribosome-binding regions of the *psaL, psaJ, chlN*, and *cpcA* mRNA and sRNA PsrR1 by the combination of the CopraRNA tool and experimental analysis.

### Microarray Analysis

Microarray analysis is the approach that uses microchips conjugated with probes (short DNA elements) for the screening of large-scale gene expression. Labeled nucleic acid samples are applied to the microchips and then hybridized to specific probes; the interaction is identified by imaging and subsequent data processing. Microarray analysis is a strong tool for the detection of sRNA, especially antisense sRNA (asRNA), whose function is mainly determined by mere sequence complementarity rather than structural characteristics or specific sequence. Since computational screens rarely find asRNAs, the superiority and preferences are important to novel sRNA by a correlation between the prediction and the actual presence of a terminator.

High-coverage genome-wide microarrays had been used to screen for the presence of sRNAs in *Prochlorococcus* MED4 and *Synechocystis* sp. PCC 6803 (Steglich et al., 2008; Georg et al., 2009). Twelve new ncRNAs and 24 asRNAs were identified though microarray analyses in *Prochlorococcus* MED4 and then 12 ncRNAs were independently verified by experiment (Steglich et al., 2008). In *Synechocystis* sp. PCC 6803, a novel transcriptome microarray was designed as an efficient tool for the verification of the possible regulation of the newly found asRNAs and ncRNAs. This array includes probe sets for all protein-coding genes as well as for all other transcripts. Totally, 11 out of 73 asRNAs and 27 out of 60 intergenic ncRNAs had been predicted with high microarray expression levels, based on the existence of a Rho-independent terminator using this approach (Georg et al., 2009). Five ncRNAs of these have been earlier reported in a comparative genomics study (Voss et al., 2009).

### RNA-Sequence Approach

Within the last few years, RNA-sequencing technology has completely changed the global identification of sRNAs in cyanobacteria and evoked a research wave in this field. As a powerful analytical tool, RNA-seq, especially differential RNA-seq (dRNA-seq), not only provides an in-depth understanding of changes in gene expression, but also the details of all promoters active at a given time, thus providing effectively an insight into the state of transcription. A dRNA-seq has established a genome-wide map of *Synechocystis* sp. PCC 6803 with 3,527 transcriptional start sites (TSSs) (Mitschke et al., 2011a). One-thirds (1,112) were found on the reverse complementary strand of 866 genes (aTSS, antisense RNA TSS), suggesting a large number of antisense transcriptions. The prediction of ncRNAs came from 429 seemingly orphan TSSs (nTSS, non-coding RNA TSS) located in intergenic regions. Genome-wide maps of active TSSs have been established and identified 4,091 transcriptional units under 10 different conditions related to photosynthetic growth (Kopf et al., 2014). Besides, several sRNAs with an intriguing expression pattern have been reported. In addition, a deep RNA-seq analysis focusing on low molecular weight RNAs (≤200 nt) predicted 5,261 and 3,380 novel transcribed sRNA regions from the genome and the four megaplasmids in *Synechocystis* sp. PCC 6803 under normal and high light conditions. Totally, 14 of these molecules, including three from different megaplasmids, were confirmed by RNA blot hybridization (Xu et al., 2014; Hu et al., 2017).

However, there are a lot of data noise and false-positives in the biocomputational prediction, tiling microarrays, or RNA-sequence approaches. So, the subsequent verification by experimental approaches is essential for the identification of sRNAs and their functional verification, e.g., RACE, Northern blot analysis, and mutant strains of overexpression/knockdown.

## Regulatory Functions of Cyanobacterial sRNAs

The sRNAs are important transcriptional and post-transcriptional regulators of gene expression in every domain of life, modulating mRNA stability, DNA maintenance, DNA silencing, transcription, and translation (Waters and Storz, 2009). They achieve these diverse outcomes by multiple mechanisms, such as changes in protein binding, base pairing with other RNAs, and interactions with DNA.

### sRNAs That Modulate Protein Activity

A small class of sRNA would interact with special target protein in order to function. Three classes of the well-studied sRNAs of those have the typical characteristics of protein-binding sRNA. The RNase P RNA has intrinsic activity, e.g., the RNase P RNA is required for survival in *Synechocystis* sp. PCC 6803 (Tous et al., 2001), while 4.5S RNA and tmRNA provide basic functions for a ribonucleoprotein particle. In contrast, another protein-binding sRNA (6S RNA) acts in a regulation to antagonize cognate proteins activities by mimicking other nucleic acids structures (Banta et al., 1992; Watanabe et al., 1997, 1998; Sugita et al., 2007). In *Synechocystis* sp. PCC 6803, nTSS (sRNA TSS) for 4.5S RNA (*ffs*), 6S RNA (*ssaA*), RNase P RNA (*rnpB*), and tmRNA (*ssrA*) were consistent with the published experimental data or genome an notation (Mitschke et al., 2011a).

The >100 new 6S RNA homologs were computationally identified in diverse eubacterial lineages and their conserved features suggested that they bind to RNA polymerase by mimicking the DNA template structure in an open promoter complex (Barrick et al., 2005). In 1997, 6Sa RNA was first isolated as a novel sRNA using an elaborate biochemical protocol from the unicellular cyanobacterium *Synechococcus* PCC 6301 (Watanabe et al., 1997). The accumulation of 6Sa RNA was observed in all cell stages and its level was significantly decreased in the stationary phase, which suggests that 6Sa RNA plays a role in cell division. Later, 6Sa RNA was recognized as 6S RNA homologs by GenBank (Barrick et al., 2005). Axmann et al. (2007) reported that two distinct types of 6S RNAs were accumulated and regulated during growth and the diel cycle in *Prochlorococcus* MED4. In order to control the timing of cell division and rhythmic regulation of gene expression, these 6S RNAs may switch between two different conformations after binding to *trans*-acting proteins or other unknown factors (Nair et al., 2002; Ditty et al., 2003; Axmann et al., 2007).

Ribonuclease P (RNase P) is a universal enzyme necessary for tRNA maturation. In cyanobacteria, the enzyme is composed of a protein subunit by *rnpA* and an RNA subunit by *rnpB*. The RNA subunit was the catalytic component of the enzyme and can precisely perform endonucleolytic cleavage of tRNA precursors to produce a mature 5′ end in the absence of the protein subunit (Frank and Pace, 1998; Tous et al., 2001; Schon et al., 2002). In *Synechocystis*, for maturation of the dimeric precursors, it needed to be cleaved at two positions by RNase P, corresponding to the mature 5′ ends of tRNA^Tyr^ and tRNA^Thr^, respectively (Kaneko and Tabata, 1997; Tous et al., 2001). Besides, RNase P was also involved in the 5′ processing of some stable RNAs, e.g., 4.5S RNA and tmRNA (Tous et al., 2001).

The tmRNA, which has been named to reflect its tRNA-like and mRNA-like properties, was first found in *E. coli* (Ray and Apirion, 1979) and then reported in many other bacteria (Brown et al., 1990; Tyagi and Kinger, 1992; Ushida et al., 1994; Fleischmann et al., 1995; Williams and Bartel, 1996). Watanabe et al. (1998) reported the first evidence of tmRNA existence in cyanobacteria, and its coding gene was temporarily named as *ssrA*. This study suggested that tmRNA functioned not only when cells divided actively, but also when cells growth stopped by reason of a fairly high level of tmRNA throughout the cell cycle. Although it has not experimentally analyzed the *Prochlorococcus* tmRNA so far, it would consist of two separate molecules from a common precursor *in silico* analyses (Keiler et al., 2000; Gaudin et al., 2002; Axmann et al., 2005).

### *Cis*-Encoded Base Pairing sRNAs

Different from the protein-binding sRNAs mentioned earlier, most characterized sRNAs have complete complementarity or limited complementarity with their target mRNA by base pairing to regulate gene expression (Waters and Storz, 2009). *Cis*-encoded asRNAs are transcribed from the opposite strand of the target gene and share complete complementarity with target mRNA. Most asRNAs reported are heavy regulatory elements and commonly encoded in accessory elements such as transposons and plasmids in bacteria (Wagner et al., 2002; Brantl, 2007). In cyanobacteria, all asRNAs found to date are chromosomally encoded RNAs (**Table 2**). *Cis*-encoded asRNAs has a great advantage in a number of cyanobacteria, for example, asRNAs amounting to 26% of all genes were reported in *Synechocystis* sp. PCC 6803 (Georg et al., 2009; Mitschke et al., 2011a) and 39% in *Anabaena* sp. PCC 7120 (Mitschke et al., 2011b). Thus, *cis*-encoded chromosomal asRNAs may have an important role in cyanobacterial regulatory networks. In cyanobacteria, *cis*-encoded asRNA represent the most investigated class of sRNA by far. However, the list of asRNAs is far from complete, especially for detailed mechanisms.

**Table 2:** *Cis*-encoded antisense sRNA indentified in cyanobacteria.

sRNA	TSS	Length	Target gene	Function	Species	Source
IsrR	1518034	177 nt	*isiA*	The protection of the PSI complex against photo-induced damage	*Synechocystis* sp. PCC 6803	Dühring et al., 2006
*α-fur* RNA	2018583	2200 nt	*α-fur-alr1690*	Regulating photosynthetic machinery	Anabaena sp. PCC 7120, conserved in cyanobacteria	Hernández et al., 2006
PsbA2R, PsbA3R	7210, 1820016	130 and 220 nt, 160 and 180 nt	*psbA2*, *psbA3*	Achieving a maximum level of D1 synthesis	*Synechocystis* sp. PCC 6803	Sakurai et al., 2012
RblR	c2478718	113 nt	*rbcL*	Regulating photosynthetic machinery	*Synechocystis* sp. PCC 6803	Hu et al., 2017
*gvp* asRNA	56 bp upstream from the start codon of the *gvpA* gene	About 420 nt	*gvpABC* operon	Involved in the formation of gas vesicles	*Calothrix* sp. PCC 7601	Csiszàr et al., 1987
As1_flv4	166849	500 and 280 nt	*flv4-2* operon	Photoprotection of photosystem II under low carbon conditions	*Synechocystis* sp. PCC 6803	Eisenhut et al., 2012
CoaR	2867076	124 nt	*slr0847*-*slr0848* operon	Regulating CoA synthesis and 1-butanol tolerance	*Synechocystis* sp. PCC 6803	Sun et al., 2017
PilR	758305	210 nt	*pilA9-pilA10-pilA11-slr2018* operon	Controlling *pilA11* gene expression and cell motility	*Synechocystis* sp. PCC 6803	Hu et al., 2018

*TSS, transcription start sites; nt, nucleotides.*

Most of *cis*-encoded chromosomal asRNAs reported so far regulate essential genes expression in response to environmental stresses, optimizing gene function and cell economy. A *cis*-encoded asRNA IsrR (iron stress-repressed RNA), which is the first well studied, was reported to play a role in protecting PSI complex from photo-induced damage in *Synechocystis* sp. PCC 6803 (Dühring et al., 2006). The IsrR RNA regulates the expression of target gene *isiA*, which forms a supercomplex around PSI under three different stress conditions: oxidative stress, high light, and iron limitation. Analysis clearly showed that IsrR overexpression strongly diminishes the IsiA-PSI supercomplexes number under iron stress, whereas isrR depletion leads to premature *isiA* expression. The IsrR appears to be expressed constitutively under moderate iron starvation to suppress *isiA* expression. In this case, IsrR filters out transient environmental stress signals and results in a delayed *isiA* expression in the early phase, but it is depleted more quickly during the stress recovery (Legewie et al., 2008).

An asRNA (*α-furA* RNA) has a complete *furA* mRNA coding sequence in the nitrogen-fixing *Anabaena* sp. PCC 7120, which transcribed in the same transcriptional level as *alr1690* (Hernández et al., 2006). Disruption of the *α-furA-alr1690* mRNA increases the level of FurA protein and the resulting mutant shows an iron-deficient phenotype, indicating the biological correlation of this asRNA (Hernandez et al., 2010). Meanwhile, in *Microcystis aeruginosa* PCC 7806 and *Synechocystis* sp. PCC 6803, it has identified other three anti-*fur* RNAs (Martin-Luna et al., 2011; Sevilla et al., 2011), indicating that anti-*fur* RNAs are highly homologous in cyanobacteria.

The PsbA2R and PsbA3R, two asRNAs located in the 5′ untranslated region (5′UTR) of target genes *psbA2* and *psbA3*, encode D1 protein of photosystem II in the thylakoid membrane of *Synechocystis* sp. PCC 6803 (Sakurai et al., 2012). The expression of PsbA2R and PsbA3R is shown to be upregulated by light and downregulated by darkness, similar to the expression of their target mRNA, and acts as a positive regulator of cell growth. These results of the PsbA2R(-) mutant strain showed that PsbA2R was a positive factor and could achieve a maximum level of D1 synthesis under some environmental conditions.

The RuBisCO catalyzes carbon fixation and is the most abundant protein in leaves, summing up to 50% of the soluble leaf protein in C3 plants and to 30% in C4 plants (Feller et al., 2008). The large chain of RuBisCO is encoded by the *rbcL* gene in cyanobacteria and land plant (Spreitzer and Salvucci, 2002). In our previous study, the 113 nt asRNA RblR that is completely complementary to its target gene *rbcL* was identified in *Synechocystis* sp. PCC 6803 (Hu et al., 2017). Analysis clearly showed that RblR has a positive regulation on *rbcL* gene expression under several stress conditions and suppressing RblR adversely affects carbon assimilation.

**FIGURE 1:**
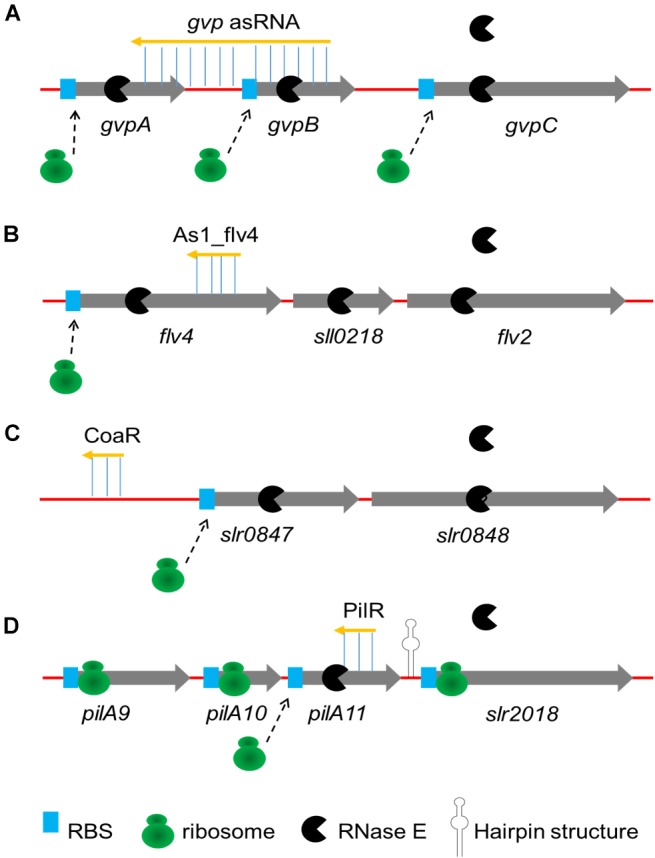
Four major mechanisms of sRNAs regulating operons. **(A)** Coordinate regulation of the *gvpABC* operon by *gvp* asRNA is performed by forming an homologous duplex with transcripts; **(B)** As1_flv4 coordinately regulates *flv4-2 operon* by binding to coding region on *flv4*, preventing translation and promoting cooperative degradation; **(C)** Coordinate regulation of the *slr0847-slr0848* operon by CoaR is performed by binding of CoaR in promotor region of *slr0847* gene; **(D)** PilR discoordinately regulates the *pilA9-pilA10-pilA11-slr2018* operon by both translational silencing and promoting degradation. A strong secondary structure in the *pilA11-slr2018* intergenic region (represented by hairpin structure) presumably prevents degradation of the *slr2018* mRNA.

The sRNA-mRNA interactions described earlier have been described one by one. Some asRNAs have been discovered to modulate the expression of genes in operons by coordinate regulation or discoordinate regulation in cyanobacteria (**Figure 1**). In some cases, the activity of sRNA activates or suppresses all genes in the operon at the same time (coordinate regulation). The first reported asRNA is a *cis*-encoded chromosomal asRNA, which is complementary to the *gvpABC* operon and involved in gas vesicles formation in *Calothrix* sp. PCC 7601 (Csiszàr et al., 1987). Therefore, this asRNA can form a homologous duplex with three transcripts of the *gvpABC* operon, which can damage translation and/or modify mRNA stability. Another *cis*-asRNA As1_flv4, derived from the *flv4-2* operon, could be transiently expressed in *Synechocystis* sp. PCC 6803 under the limitation of inorganic carbon (Ci) (Eisenhut et al., 2012). During the early phase of low carbon acclimation, As1_flv4 plays roles in Ci-dependent regulation of Flv4-2 proteins by establishing safety threshold and transferring expression in time. The respective asRNA of the *flv4-2* operon acts as a buffer mechanism to avoid premature operon expression under stress (Zhang et al., 2009, 2012). The CoaR is a negative regulator of *slr0847* (coaD) and *slr0848* operon, which is responsible for the synthesis of coenzyme A (CoA) and may regulate the tolerance to 1-butanol by downregulating the synthesis of CoA, thus reducing fatty acid metabolism and energy metabolism (Sun et al., 2017). While in other cases, sRNAs are not coupled with cistrons expression, resulting in uncoordinated regulation of the operon. Cell motility relies on the expression of putative *pilA9-pilA10-pilA11-slr2018* operon in *Synechocystis* sp. PCC 6803 (Panichkin et al., 2006). Recently, an asRNA PilR was identified in *Synechocystis* sp. PCC 6803, and it encoded in the non-coding strand of the prepilin-encoding gene *pilA11* (Hu et al., 2018). The PilR reduced the expression levels of *pilA11*, but no other gene in the *pilA9-pilA10-pilA11-slr2018* operon, while reducing both the number and thickness of pili, limiting the motility of cells.

### *Trans*-Encoded Base Pairing sRNAs

In cyanobacteria, in contrast to *cis*-encoded asRNAs, several of which have an in-depth study, another class of incomplete base-pairing sRNAs is the *trans*-encoded sRNAs (**Table 3**). Sequence comparison of intergenic regions has revealed several *trans*-encoded sRNAs Yfr in several different cyanobacteria of the *Prochlorococcus-Synechococcus* lineage (Axmann et al., 2005). Of these, the Yfr2 sRNAs family (Yfr2-5) is a group of abundant RNA molecules and widely present in nearly all cyanobacteria sequenced to date (Gierga et al., 2009).

**Table 3:** *Trans*-encoded sRNAs indentified in cyanobacteria.

sRNA	TSS	Length	target gene	Function	Species	Source
Yfr1	2417299	65 nt	*sbtA*	Maintenance of normal growth under various stress conditions	*Synechococcus elongatus* PCC 6301	Nakamura et al., 2007
SyR1/PsrR1	1671919	131 nt	*psaL, psaJ, chlN, and cpcA*	Controlling photosynthetic functions	*Synechocystis* sp. PCC 6803, conserved in cyanobacteria	Georg et al., 2014
IsaR1	3164320	68 nt	More than 15 direct targets, including Fe^2+^-containing proteins involved in photosynthetic electron transfer, detoxification of anion radicals, citrate cycle, and tetrapyrrole biogenesis	Acclimating photosynthetic apparatus to iron starvation	*Synechocystis* sp. PCC 6803, conserved in cyanobacteria	Georg et al., 2017
Nc117	3250530	102 nt	*slr0007*	Positively regulating biofuels tolerance	*Synechocystis* sp. PCC 6803	Pei et al., 2017; Bi et al., 2018

*TSS, transcription start sites; nt, nucleotides.*

For *trans*-encoded sRNAs, the chromosomal location of the sRNA gene has little correlation with the target mRNA gene. The *yfr1* gene, a high conserved gene found in various cyanobacterial strains, is 65 nucleotides long and positioned between *guaB* and *trxA* in *Synechococcus elongatus* PCC 6301 (genome position: 2,417,299–2,417,363). However, the target *sbtA* transcript of Yfr1 has a distant position (genome position: 2,623,057–2,624,178) (Nakamura et al., 2007). Furthermore, the *yfr1*-disruption mutant, showed growth defects under various stress conditions.

In contrast to the *cis*-encoded asRNAs, the *trans*-encoded sRNAs modulate more than one target by formation of partial RNA-RNA duplexes. These sRNAs control the stability and translation of target mRNAs and have similar functions to eukaryotic miRNAs in many respects (Gottesman, 2005; Aiba, 2007). By computational and experimental data, sRNA PsrR1 has interaction with the ribosome-binding regions of the *psaL, psaJ, chlN*, and *cpcA* mRNA in *Synechocystis* sp. PCC 6803 (Georg et al., 2014). As a regulatory factor for photosynthetic functions, sRNA PsrR1 and the transcriptional regulator RpaB constituted a feed-forward loop to regulate PSI gene expression to achieve a rapid adaptive response to the high light stress condition (Kadowaki et al., 2016). Oxygenic photosynthesis depends to a large extent on proteins that contain Fe^2+^ or Fe/S complexes as cofactors or prosthetic groups (Keren et al., 2004). Georg et al. showed that sRNA IsaR1 was induced by iron starvation and then acted on photosynthetic apparatus in three specific ways. The IsaR1 had more than 15 direct targets and controlled a complex network to acclimate to low iron condition (Georg et al., 2017). A *trans*-encoded sRNA Nc117 was discovered involved in the tolerance of *Synechocystis* sp. PCC 6803 to ethanol and 1-butanol (Pei et al., 2017). A total of 119 upregulated and 116 downregulated genes were identified by comparative transcriptomic analysis, among which a gene *slr0007* encoding D-glycero-alpha-D-mannoheptose 1-phosphate guanylyltransferase was determined to be a potential target of Nc117. These results suggested that the synthesis of lipopolysaccharide or S-layer glycoprotein may be related to the increased tolerance (Bi et al., 2018).

### Hfq Protein of RNA Chaperone

In *E. coli* and closely related bacteria, the RNA chaperone Hfq usually participates in *trans*-encoded sRNA-mediated regulation, presumably to facilitate sRNA-mRNA duplex formation (Valentin-Hansen et al., 2004; Aiba, 2007; Brennan and Link, 2007). Several studies have found that hexameric Hfq ring was homologous to Sm and Sm-like proteins involved in mRNA decay and splicing in eukaryotes and archaea (Valentin-Hansen et al., 2004; Nielsen et al., 2007). In cyanobacteria, preliminary attempts to explore microbial genomes of Hfq homologs failed (Sun et al., 2002). However, combined with motif and pattern sequence searches, Hfq orthologs from various cyanobacteria were identified (Valentin-Hansen et al., 2004). The cyanobacterial Hfq homolog is proved to be essential for motility of *Synechocystis* sp. PCC 6803 through the knockout of *hfq* (Dienst et al., 2008). The crystal structures of Hfq-homolog proteins retained the classic Sm folding despite low-sequence conservation in *Synechocystis* sp. PCC 6803 and *Anabaena* sp. PCC 7120 (Bøggild et al., 2009), suggesting the higher conservation of Hfq proteins in bacteria than initially anticipated (**Table 4**).

**Table 4:** Hfq protein of RNA chaperone in cyanobacteria.

RNA chaperone	Characteristic	Species	Source
Hfq	Essential for cell motility	*Synechocystis* sp. PCC 6803	Dienst et al., 2008
Hfq	Retaining the classic Sm fold	*Synechocystis* sp. PCC 6803, *Anabaena* sp. PCC 7120	Bøggild et al., 2009

On the contrary, Hfq and homologue proteins are not ubiquitous in all of cyanobacteria. The *hfq* has been lost in several sequenced *Prochlorococcus* strains, e.g., *Prochlorococcus* MED4 (Axmann et al., 2005; Steglich et al., 2008). The naturally *hfq*-deficient *Prochlorococcus* MED4 expresses 24 different sRNAs (Axmann et al., 2005; Steglich et al., 2008). The phenomenon suggests that a class of sRNAs functions that do not require partner support of a chaperone or have a novel chaperone are yet to be identified.

## Physiological Roles and Functions of sRNA

Although there has not been much research done, many sRNAs studied possessed important physiological roles and are related to environmental stresses, photosynthesis, CO_2_ fixation, and metabolism in the cyanobacteria (**Table 5**). Especially, *trans*-coded sRNA and some *cis*-encoded asRNA respond generally to adverse environmental conditions by regulating metabolic pathways or alarm reaction. The asRNAs largely function against various environmental insults by the synthesis of target proteins, e.g., the target protein levels of IsiA (Dühring et al., 2006), Fur (Martin-Luna et al., 2011), PsbA2 and PsbA3 (Sakurai et al., 2012), RbcL (Hu et al., 2017), CoaD (Sun et al., 2017), and Flv4-2 (Eisenhut et al., 2012) are influenced by corresponding asRNA in iron-, light-, oxidative-, 1-butanol-stress, and carbon-limited conditions. Moreover, some sRNAs are required for growth under multiple stress conditions, e.g., the *Synechococcus elongatus* Yfr1, which has homologs in other cyanobacteria (Nakamura et al., 2007; Voss et al., 2007). The 14 new sRNA and 24 asRNA had been identified from intergenic regions of *Prochlorococcus* MED4 under various different stress conditions (Steglich et al., 2008). Some of these sRNAs expression profiles suggested involvement in bacteriophage infection response and/or light stress adaptation.

**Table 5:** Physiological roles and functions of sRNAs in cyanobacteria.

sRNA	Target gene	Physiological roles	Species	Source
IsrR	*isiA*	Response to iron, light, and oxidative stress	*Synechocystis* sp. PCC 6803	Dühring et al., 2006
*α-fur* RNA	*α-fur-alr1690*	Response to light and oxidative stress	*Microcystis aeruginosa* PCC 7806	Martin-Luna et al., 2011
PsbA2R and PsbA3R	*psbA2* and *psbA3*	Response to light stress	*Synechocystis* sp. PCC 6803	Sakurai et al., 2012
RblR	*rbcL*	Response to light and carbon-limited stress	*Synechocystis* sp. PCC 6803	Hu et al., 2017
CoaR	*coaD*-*slr0848* operon	Response to 1-butanol stress	*Synechocystis* sp. PCC 6803	Sun et al., 2017
As1_flv4	*flv4-2* operon	Response to carbon-limited stress	*Synechocystis* sp. PCC 6803	Eisenhut et al., 2012
Yfr1	*sbtA*	Response to various stress, e.g., oxidative, high salt, Fe^2+^ limitation, and calcium limitation stress	*Prochlorococcus/Synechococcus*, conserved in cyanobacteria	Nakamura et al., 2007; Voss et al., 2007
14 New ncRNAs		Response to light qualities and quantities, phage, and phosphorus starvation stress	*Prochlorococcus* MED4	Steglich et al., 2008

Cyanobacteria are usually obedient organisms for genetic manipulation, but the lack of powerful metabolic engineering tools has seriously restricted the attempts to implement more complex genetic regulation (Huang et al., 2010). In recent years, much attention has been paid to genetic tools based on artificial sRNAs as a result of their obvious superiority (Zess et al., 2016; Sun et al., 2018). Firstly, sRNA regulatory tools mostly do not impose any metabolic burden on host cells (Gaida et al., 2013). Secondly, the tunable base-pair complementation sRNAs permit whole genome regulation of target genes for fine flux control (Rodrigo et al., 2012; Na et al., 2013). The last but not the least, sRNA regulatory tools could effectively knock the essential genes from the host cell, in contrast to the traditional lethal knockout method of these genes (Nakashima and Tamura, 2009). On the basis of interaction between sRNAs and their target mRNAs, some physiological and metabolic pathways of cyanobacteria can be modulated and altered by artificial sRNA tools. A *trans*-encoded sRNA system was constructed and adapted in *Synechococcus* sp. PCC 7002 (Zess et al., 2016). This system exhibited 70% depression of target gene expression, demonstrating sRNA regulatory tools for differential gene expression in cyanobacteria. Moreover, two sRNA regulatory tools were reported in *Synechocystis* sp. PCC 6803, which manipulated basic metabolic pathways and redirected carbon flux, based on a exogenous Hfq chaperone and a well-studied MicC scaffold previously developed in *E. coli* (Sun et al., 2018). For regulating multiple genes simultaneously and modifying into an inducible system, the Hfq-MicC tool was developed based on the theophylline-induced riboswitch. The sRNA regulatory tools introduce efficient and valuable metabolic and physiological regulatory strategies for cyanobacteria and, therefore, have showed promising applications.

## Prospect

In recent years, approaches for sRNAs detecting and targeting have become a standard method used to search for several types of sRNA genes within bacterial genomes. Meanwhile, many target prediction tools and related software of bacteria have been developed. The sRNA regulatory systems are identified as efficient and valuable physiological regulatory strategies in cyanobacteria. Therefore, cyanobacterial sRNAs are well worth exploring as a strong and facile regulatory factor in future.

At present, RNA molecules hold many types of regulatory functions in bacteria and control almost all aspects of cell metabolism. A large number of sRNAs are intriguing as they may represent a mode of adaption under various environmental conditions. Regulation by sRNAs may require fewer resources than protein regulators synthesis (Shimoni et al., 2007; Mehta et al., 2008). Furthermore, there might be positive selection pressure to maintain small regulators rather than large protein regulators during genome reduction. Outstanding progress has been made in the identification and characterization of cyanobacterial sRNAs, indicating clearly that RNA regulators are ubiquitous and often conservative, possibly exceeding the number and diversity of protein regulators. However, many exciting questions about mechanism and function of some sRNAs still remain. The current focus is on developing tools to correctly predict sRNA targets. Several target prediction tools have been developed, but there are still considerable developments to be made. The identification of sRNA targets would allow the unraveling of pathways that involve sRNA-induced regulation. It would also be a further important step for the functional characterization of the many found sRNAs.

## Author Contributions

JH and QW gathered the information and wrote and edited the manuscript. QW revised and approved the work.

## Conflict of Interest Statement

The authors declare that the research was conducted in the absence of any commercial or financial relationships that could be construed as a potential conflict of interest.

## Abbreviations

nt: nucleotides
sRNA: small RNA

## References

[B1] Abed R. M. Dobretsov S. Sudesh K. (2009). Applications of cyanobacteria in biotechnology. *J. Appl. Microbiol.* 106 1–12. 10.1111/j.1365-2672.2008.03918.x 19191979

[B2] Aiba H. (2007). Mechanism of RNA silencing by Hfq-binding small RNAs. *Curr. Opin. Microbiol.* 10 134–139. 10.1016/j.mib.2007.03.010 17383928

[B3] Axmann I. M. Holtzendorff J. Voss B. Kensche P. Hess W. R. (2007). Two distinct types of 6S RNA in *Prochlorococcus*. *Gene* 406 69–78. 10.1016/j.gene.2007.06.011 17640832

[B4] Axmann I. M. Kensche P. Vogel J. Kohl S. Herzel H. Hess W. R. (2005). Identification of cyanobacterial non-coding RNAs by comparative genome analysis. *Genome Biol.* 6:R73. 10.1186/gb-2005-6-9-r73 16168080 PMC1242208

[B5] Backofen R. Hess W. R. (2010). Computational prediction of sRNAs and their targets in bacteria. *RNA Biol.* 7 33–42. 10.4161/rna.7.1.10655 20061798

[B6] Banta A. B. Haas E. S. Brown J. W. Pace N. R. (1992). Sequence of the ribonuclease P RNA gene from the cyanobacterium *Anacystis nidulans*. *Nucleic Acids Res.* 20:911 10.1093/nar/20.4.911 PMC312038 1371871

[B7] Barrick J. E. Sudarsan N. Weinberg Z. Ruzzo W. L. Breaker R. R. (2005). 6S RNA is a widespread regulator of eubacterial RNA polymerase that resembles an open promoter. *RNA* 11 774–784. 10.1261/rna.7286705 15811922 PMC1370762

[B8] Bi Y. Pei G. Sun T. Chen Z. Chen L. Zhang W. (2018). Regulation mechanism mediated by trans-encoded sRNA Nc117 in short chain alcohols tolerance in *Synechocystis* sp. PCC 6803. *Front. Microbiol.* 9:863. 10.3389/fmicb.2018.00863 29780373 PMC5946031

[B9] Bøggild A. Overgaard M. Valentin-Hansen P. Brodersen D. E. (2009). Cyanobacteria contain a structural homologue of the Hfq protein with altered RNA-binding properties. *FEBS J.* 276 3904–3915. 10.1111/j.1742-4658.2009.07104.x 19777643

[B10] Brantl S. (2007). Regulatory mechanisms employed by cis-encoded antisense RNAs. *Curr. Opin. Microbiol.* 10 102–109. 10.1016/j.mib.2007.03.012 17387036

[B11] Brennan R. G. Link T. M. (2007). Hfq structure, function and ligand binding. *Curr. Opin. Microbiol.* 10 125–133. 10.1016/j.mib.2007.03.015 17395525

[B12] Brown J. W. Hunt D. A. Pace N. R. (1990). Nucleotide sequence of the 10Sa RNA gene of the beta-purple eubacterium *Alcaligenes eutrophus*. *Nucleic Acids Res.* 18:2820. 10.1093/nar/18.9.2820 1692615 PMC330778

[B13] Busch A. Richter A. S. Backofen R. (2008). IntaRNA: efficient prediction of bacterial sRNA targets incorporating target site accessibility and seed regions. *Bioinformatics* 24 2849–2856. 10.1093/bioinformatics/btn544 18940824 PMC2639303

[B14] Cao Y. Zhao Y. Cha L. Ying X. Wang L. Shao N. (2009). sRNATarget: a web server for prediction of bacterial sRNA targets. *Bioinformation* 3 364–366. 10.6026/97320630003364 19707302 PMC2720669

[B15] Chen Z. Zhan J. Chen Y. Yang M. He C. Ge F. (2015). Effects of phosphorylation of beta subunits of phycocyanins on state transition in the model cyanobacterium *Synechocystis* sp. PCC 6803. *Plant Cell Physiol.* 56 1997–2013. 10.1093/pcp/pcv118 26315596

[B16] Csiszàr K. Houmard J. Damerval T. de Marsac N. T. (1987). Transcriptional analysis of the cyanobacterial gvpABC operon in differentiated cells: occurrence of an antisense RNA complementary to three overlapping transcripts. *Gene* 60 29–37. 10.1016/0378-1119(87)90210-1 2450053

[B17] DennisG.Jr.ShermanB. T.HosackD. A.YangJ.GaoW.LaneH. C. (2003). DAVID: database for annotation, visualization, and integrated discovery. *Genome Biol.* 4:P3 10.1186/gb-2003-4-5-p312734009

[B18] Dienst D. Dühring U. Mollenkopf H. -J. Vogel J. Golecki J. Hess W. R. (2008). The cyanobacterial homologue of the RNA chaperone Hfq is essential for motility of *Synechocystis* sp. PCC 6803. *Microbiology* 154 3134–3143. 10.1099/mic.0.2008/020222-0 18832319

[B19] Ditty J. Williams S. Golden S. (2003). A cyanobacterial circadian timing mechanism. *Annu. Rev. Genet.* 37 513–543. 10.1146/annurev.genet.37.110801.142716 14616072

[B20] Dühring U. Axmann I. M. Hess W. R. Wilde A. (2006). An internal antisense RNA regulates expression of the photosynthesis gene isiA. *Proc. Natl. Acad. Sci. U.S.A.* 103 7054–7058. 10.1073/pnas.0600927103 16636284 PMC1459017

[B21] Eisenhut M. Georg J. Klahn S. Sakurai I. Mustila H. Zhang P. (2012). The antisense RNA As1_flv4 in the cyanobacterium *Synechocystis* sp. PCC 6803 prevents premature expression of the flv4-2 operon upon shift in inorganic carbon supply. *J. Biol. Chem.* 287 33153–33162. 10.1074/jbc.M112.391755 22854963 PMC3460422

[B22] Feller U. Anders I. Mae T. (2008). Rubiscolytics: fate of Rubisco after its enzymatic function in a cell is terminated. *J. Exp. Bot.* 59 1615–1624. 10.1093/jxb/erm242 17975207

[B23] Fleischmann R. D. Adams M. D. White O. Clayton R. A. Kirkness E. F. Kerlavage A. R. (1995). Whole-genome random sequencing and assembly of *Haemophilus influenzae* Rd. *Science* 269 496–512. 10.1126/science.7542800 7542800

[B24] Flores F. G. Herrero A. (2008). *The Cyanobacteria: Molecular Biology, Genomics, and Evolution.* Poole: Horizon Scientific Press.

[B25] Frank D. N. Pace N. R. (1998). Ribonuclease P: unity and diversity in a tRNA processing ribozyme. *Annu. Rev. Biochem.* 67 153–180. 10.1146/annurev.biochem.67.1.153 9759486

[B26] Gaida S. M. Al-Hinai M. A. Indurthi D. C. Nicolaou S. A. Papoutsakis E. T. (2013). Synthetic tolerance: three noncoding small RNAs, DsrA, ArcZ and RprA, acting supra-additively against acid stress. *Nucleic Acids Res.* 41 8726–8737. 10.1093/nar/gkt651 23892399 PMC3794604

[B27] Gaudin C. Zhou X. Williams K. P. Felden B. (2002). Two-piece tmRNA in cyanobacteria and its structural analysis. *Nucleic Acids Res.* 30 2018–2024. 10.1093/nar/30.9.2018 11972341 PMC113835

[B28] Georg J. Dienst D. Schurgers N. Wallner T. Kopp D. Stazic D. (2014). The small regulatory RNA SyR1/PsrR1 controls photosynthetic functions in cyanobacteria. *Plant Cell* 26 3661–3679. 10.1105/tpc.114.129767 25248550 PMC4213160

[B29] Georg J. Kostova G. Vuorijoki L. Schon V. Kadowaki T. Huokko T. (2017). Acclimation of Oxygenic Photosynthesis to Iron Starvation Is Controlled by the sRNA IsaR1. *Curr. Biol.* 27 1425.e7–1436.e7. 10.1016/j.cub.2017.04.010 28479323

[B30] Georg J. Voss B. Scholz I. Mitschke J. Wilde A. Hess W. R. (2009). Evidence for a major role of antisense RNAs in cyanobacterial gene regulation. *Mol. Syst. Biol.* 5:305. 10.1038/msb.2009.63 19756044 PMC2758717

[B31] Gierga G. Voss B. Hess W. R. (2009). The Yfr2 ncRNA family, a group of abundant RNA molecules widely conserved in cyanobacteria. *RNA Biol.* 6 222–227. 10.4161/rna.6.3.8921 19502815

[B32] Giordano M. Wang Q. (2018). “Microalgae for industrial purposes,” in *Biomass and Green Chemistry: Building a Renewable Pathway*, ed. Vaz S. Jr (Cham: Springer International Publishing), 133–167.

[B33] Goebel N. L. Turk K. A. Achilles K. M. Paerl R. Hewson I. Morrison A. E. (2010). Abundance and distribution of major groups of diazotrophic cyanobacteria and their potential contribution to N(2) fixation in the tropical Atlantic Ocean. *Environ. Microbiol.* 12 3272–3289. 10.1111/j.1462-2920.2010.02303.x 20678117

[B34] Gottesman S. (2005). Micros for microbes: non-coding regulatory RNAs in bacteria. *Trends Genet.* 21 399–404. 10.1016/j.tig.2005.05.008 15913835

[B35] Hernandez J. A. Alonso I. Pellicer S. Luisa Peleato M. Cases R. Strasser R. J. (2010). Mutants of Anabaena sp. PCC 7120 lacking alr1690 and alpha-furA antisense RNA show a pleiotropic phenotype and altered photosynthetic machinery. *J. Plant Physiol.* 167 430–437. 10.1016/j.jplph.2009.10.009 19939500

[B36] Hernández J. A. Muro-Pastor A. M. Flores E. Bes M. T. Peleato M. L. Fillat M. F. (2006). Identification of a furA cis Antisense RNA in the Cyanobacterium *Anabaena* sp. PCC 7120. *J. Mol. Biol.* 355 325–334. 10.1016/j.jmb.2005.10.079 16324715

[B37] Hu J. Li T. Xu W. Zhan J. Chen H. He C. (2017). Small antisense RNA RblR positively regulates RuBisCo in *Synechocystis* sp. PCC 6803. *Front. Microbiol.* 8:231. 10.3389/fmicb.2017.00231 28261186 PMC5306279

[B38] Hu J. Zhan J. Chen H. He C. Cang H. Wang Q. (2018). The small regulatory antisense RNA PilR affects pilus formation and cell motility by negatively regulating pilA11 in Synechocystis sp. PCC 6803. *Front. Microbiol.* 9:786. 10.3389/fmicb.2018.00786 29740417 PMC5924778

[B39] Huang D. W. Sherman B. T. Lempicki R. A. (2008). Systematic and integrative analysis of large gene lists using DAVID bioinformatics resources. *Nat. Protoc.* 4 44–57. 10.1038/nprot.2008.211 19131956

[B40] Huang H. H. Camsund D. Lindblad P. Heidorn T. (2010). Design and characterization of molecular tools for a synthetic biology approach towards developing cyanobacterial biotechnology. *Nucleic Acids Res.* 38 2577–2593. 10.1093/nar/gkq164 20236988 PMC2860132

[B41] Huttenhofer A. Brosius J. Bachellerie J. P. (2002). RNomics: identification and function of small, non-messenger RNAs. *Curr. Opin. Microbiol.* 6 835–843. 10.1016/s1367-5931(02)00397-6 12470739

[B42] Huttenhofer A. Schattner P. Polacek N. (2005). Non-coding RNAs: hope or hype? *Trends Genet.* 21 289–297. 10.1016/j.tig.2005.03.007 15851066

[B43] Hüttenhofer A. Vogel J. (2006). Experimental approaches to identify non-coding RNAs. *Nucleic Acids Res.* 34 635–646. 10.1093/nar/gkj469 16436800 PMC1351373

[B44] Kadowaki T. Nagayama R. Georg J. Nishiyama Y. Wilde A. Hess W. R. (2016). A feed-forward loop consisting of the response regulator RpaB and the small RNA PsrR1 controls light acclimation of photosystem i gene expression in the cyanobacterium *Synechocystis* sp. PCC 6803. *Plant Cell Physiol.* 57 813–823. 10.1093/pcp/pcw028 26872833

[B45] Kaneko T. Tabata S. (1997). Complete genome structure of the unicellular cyanobacterium *Synechocystis* sp. PCC6803. *Plant Cell Physiol.* 38 1171–1176. 10.1093/oxfordjournals.pcp.a029103 9435137

[B46] Keiler K. C. Shapiro L. Williams K. P. (2000). tmRNAs that encode proteolysis-inducing tags are found in all known bacterial genomes: a two-piece tmRNA functions in *Caulobacter*. *Proc. Natl. Acad. Sci. U.S.A.* 97 7778–7783. 10.1073/pnas.97.14.7778 10884408 PMC16621

[B47] Keren N. Aurora R. Pakrasi H. B. (2004). Critical roles of bacterioferritins in iron storage and proliferation of cyanobacteria. *Plant Physiol.* 135 1666–1673. 10.1104/pp.104.042770 15247377 PMC519080

[B48] Kopf M. Klahn S. Scholz I. Matthiessen J. K. Hess W. R. Voss B. (2014). Comparative analysis of the primary transcriptome of *Synechocystis* sp. PCC 6803. *DNA Res.* 21 527–539. 10.1093/dnares/dsu018 24935866 PMC4195498

[B49] Landt S. G. Abeliuk E. McGrath P. T. Lesley J. A. McAdams H. H. Shapiro L. (2008). Small non-coding RNAs in *Caulobacter crescentus*. *Mol. Microbiol.* 68 600–614. 10.1111/j.1365-2958.2008.06172.x 18373523 PMC7540941

[B50] Lee E. J. Groisman E. A. (2010). An antisense RNA that governs the expression kinetics of a multifunctional virulence gene. *Mol. Microbiol.* 76 1020–1033. 10.1111/j.1365-2958.2010.07161.x 20398218 PMC2909850

[B51] Legewie S. Dienst D. Wilde A. Herzel H. Axmann I. M. (2008). Small RNAs establish delays and temporal thresholds in gene expression. *Biophys. J.* 95 3232–3238. 10.1529/biophysj.108.133819 18599624 PMC2547459

[B52] Livny J. Teonadi H. Livny M. Waldor M. K. (2008). High-throughput, kingdom-wide prediction and annotation of bacterial non-coding RNAs. *PLoS One* 3:e3197. 10.1371/journal.pone.0003197 18787707 PMC2527527

[B53] Livny J. Waldor M. K. (2007). Identification of small RNAs in diverse bacterial species. *Curr. Opin. Microbiol.* 10 96–101. 10.1016/j.mib.2007.03.005 17383222

[B54] Lott S. C. Schafer R. A. Mann M. Backofen R. Hess W. R. Voss B. (2018). GLASSgo - automated and reliable detection of sRNA homologs from a single input sequence. *Front. Genet.* 9:124. 10.3389/fgene.2018.00124 29719549 PMC5913331

[B55] Ma F. Zhang X. Zhu X. Li T. Zhan J. Chen H. (2017). Dynamic changes of IsiA-containing complexes during long-term iron deficiency in *Synechocystis* sp. PCC 6803. *Mol. Plant* 10 143–154. 10.1016/j.molp.2016.10.009 27777125

[B56] Mandin P. Repoila F. Vergassola M. Geissmann T. Cossart P. (2007). Identification of new noncoding RNAs in *Listeria monocytogenes* and prediction of mRNA targets. *Nucleic Acids Res.* 35 962–974. 10.1093/nar/gkl1096 17259222 PMC1807966

[B57] Martin-Luna B. Sevilla E. Gonzalez A. Bes M. T. Fillat M. F. Peleato M. L. (2011). Expression of fur and its antisense alpha-fur from *Microcystis aeruginosa* PCC7806 as response to light and oxidative stress. *J. Plant Physiol.* 168 2244–2250. 10.1016/j.jplph.2011.08.006 21940066

[B58] Mattick J. S. (2001). Non-coding RNAs: the architects of eukaryotic complexity. *EMBO Rep.* 2 986–991. 10.1093/embo-reports/kve230 11713189 PMC1084129

[B59] Mehta P. Goyal S. Wingreen N. S. (2008). A quantitative comparison of sRNA-based and protein-based gene regulation. *Mol. Syst. Biol.* 4:221. 10.1038/msb.2008.58 18854820 PMC2583084

[B60] Mitschke J. Georg J. Scholz I. Sharma C. M. Dienst D. Bantscheff J. (2011a). An experimentally anchored map of transcriptional start sites in the model cyanobacterium *Synechocystis* sp. PCC6803. *Proc. Natl. Acad. Sci. U.S.A.* 108 2124–2129. 10.1073/pnas.1015154108 21245330 PMC3033270

[B61] Mitschke J. Vioque A. Haas F. Hess W. R. Muro-Pastor A. M. (2011b). Dynamics of transcriptional start site selection during nitrogen stress-induced cell differentiation in Anabaena sp. PCC7120. *Proc. Natl. Acad. Sci. U.S.A.* 108 20130–20135. 10.1073/pnas.1112724108 22135468 PMC3250118

[B62] Mizuno T. Chou M. Y. Inouye M. (1984). A unique mechanism regulating gene expression: translational inhibition by a complementary RNA transcript (micRNA). *Proc. Natl. Acad. Sci. U.S.A.* 81 1966–1970. 10.1073/pnas.81.7.1966 6201848 PMC345417

[B63] Muckstein U. Tafer H. Hackermuller J. Bernhart S. H. Stadler P. F. Hofacker I. L. (2006). Thermodynamics of RNA-RNA binding. *Bioinformatics* 22 1177–1182. 10.1093/bioinformatics/btl024 16446276

[B64] Na D. Yoo S. M. Chung H. Park H. Park J. H. Lee S. Y. (2013). Metabolic engineering of *Escherichia coli* using synthetic small regulatory RNAs. *Nat. Biotechnol.* 31 170–174. 10.1038/nbt.2461 23334451

[B65] Nair U. Ditty J. L. Min H. Golden S. S. (2002). Roles for sigma factors in global circadian regulation of the cyanobacterial genome. *J. Bacteriol.* 184 3530–3538. 10.1128/JB.184.13.3530-3538.2002 12057947 PMC135120

[B66] Nakamura T. Naito K. Yokota N. Sugita C. Sugita M. (2007). A cyanobacterial non-coding RNA, Yfr1, is required for growth under multiple stress conditions. *Plant Cell Physiol.* 48 1309–1318. 10.1093/pcp/pcm098 17664182

[B67] Nakashima N. Tamura T. (2009). Conditional gene silencing of multiple genes with antisense RNAs and generation of a mutator strain of *Escherichia coli*. *Nucleic Acids Res.* 37:e103. 10.1093/nar/gkp498 19515932 PMC2731896

[B68] Nielsen J. S. Bøggild A. Andersen C. B. Nielsen G. Boysen A. Brodersen D. E. (2007). An Hfq-like protein in archaea: crystal structure and functional characterization of the Sm protein from *Methanococcus jannaschii*. *RNA* 13 2213–2223. 10.1261/rna.689007 17959927 PMC2080587

[B69] Panichkin V. B. Arakawa-Kobayashi S. Kanaseki T. Suzuki I. Los D. A. Shestakov S. V. (2006). Serine/threonine protein kinase SpkA in *Synechocystis* sp. strain PCC 6803 is a regulator of expression of three putative pilA operons, formation of thick pili, and cell motility. *J. Bacteriol.* 188 7696–7699. 10.1128/JB.00838-06 16916897 PMC1636250

[B70] Park S.-Y. Cromie M. J. Lee E.-J. Groisman E. A. (2010). A bacterial mRNA leader that employs different mechanisms to sense disparate intracellular signals. *Cell* 142 737–748. 10.1016/j.cell.2010.07.046 20813261 PMC2967377

[B71] Pei G. Sun T. Chen S. Chen L. Zhang W. (2017). Systematic and functional identification of small non-coding RNAs associated with exogenous biofuel stress in cyanobacterium *Synechocystis* sp. PCC 6803. *Biotechnol. Biofuels* 10:57. 10.1186/s13068-017-0743-y 28286552 PMC5341163

[B72] Pisciotta J. M. Zou Y. Baskakov I. V. (2010). Light-dependent electrogenic activity of cyanobacteria. *PLoS One* 5:e10821. 10.1371/journal.pone.0010821 20520829 PMC2876029

[B73] Quintana N. Kooy F. Rhee M. Voshol G. Verpoorte R. (2011). Renewable energy from Cyanobacteria: energy production optimization by metabolic pathway engineering. *Appl. Microbiol. Biotechnol.* 91 471–490. 10.1007/s00253-011-3394-0 21691792 PMC3136707

[B74] Raghavan R. Groisman E. A. Ochman H. (2011). Genome-wide detection of novel regulatory RNAs in *E. coli*. *Genome Res.* 21 1487–1497. 10.1101/gr.119370.110 21665928 PMC3166833

[B75] Ray B. K. Apirion D. (1979). Characterization of 10S RNA: a new stable RNA molecule from *Escherichia coli*. *Mol. Gen. Genet.* 174 25–32. 10.1007/BF00433301 384159

[B76] Rehmsmeier M. Steffen P. Hochsmann M. Giegerich R. (2004). Fast and effective prediction of microRNA/target duplexes. *RNA* 10 1507–1517. 10.1261/rna.5248604 15383676 PMC1370637

[B77] Rodrigo G. Landrain T. E. Jaramillo A. (2012). De novo automated design of small RNA circuits for engineering synthetic riboregulation in living cells. *Proc. Natl. Acad. Sci. U.S.A.* 109 15271–15276. 10.1073/pnas.1203831109 22949707 PMC3458397

[B78] Romby P. Vandenesch F. Wagner E. G. (2006). The role of RNAs in the regulation of virulence-gene expression. *Curr. Opin. Microbiol.* 9 229–236. 10.1016/j.mib.2006.02.005 16529986

[B79] Sakurai I. Stazic D. Eisenhut M. Vuorio E. Steglich C. Hess W. R. (2012). Positive regulation of psbA gene expression by cis-encoded antisense RNAs in *Synechocystis* sp. PCC 6803. *Plant Physiol.* 160 1000–1010. 10.1104/pp.112.202127 22858634 PMC3461525

[B80] Schon A. Fingerhut C. Hess W. R. (2002). Conserved and variable domains within divergent rnase P RNA gene sequences of *Prochlorococcus* strains. *Int. J. Syst. Evol. Microbiol.* 52(Pt 4), 1383–1389. 12148654 10.1099/00207713-52-4-1383

[B81] Schopf J. W. (1993). Microfossils of the early archean apex chert: new evidence of the antiquity of life. *Science* 260 640–646. 10.1126/science.260.5108.640 11539831

[B82] Sevilla E. Martin-Luna B. Gonzalez A. Gonzalo-Asensio J. A. Peleato M. L. Fillat M. F. (2011). Identification of three novel antisense RNAs in the fur locus from unicellular cyanobacteria. *Microbiology* 157(Pt 12), 3398–3404 10.1099/mic.0.048231-0 21921103

[B83] Shimoni Y. Friedlander G. Hetzroni G. Niv G. Altuvia S. Biham O. (2007). Regulation of gene expression by small non-coding RNAs: a quantitative view. *Mol. Syst. Biol.* 3:138. 10.1038/msb4100181 17893699 PMC2013925

[B84] Simons R. W. Kleckner N. (1983). Translational control of IS10 transposition. *Cell* 34 683–691. 10.1016/0092-8674(83)90401-4 6311438

[B85] Sittka A. Lucchini S. Papenfort K. Sharma C. M. Rolle K. Binnewies T. T. (2008). Deep sequencing analysis of small noncoding RNA and mRNA targets of the global post-transcriptional regulator, Hfq. *PLoS Genet.* 4:e1000163. 10.1371/journal.pgen.1000163 18725932 PMC2515195

[B86] Spreitzer R. J. Salvucci M. E. (2002). Rubisco: structure, regulatory interactions, and possibilities for a better enzyme. *Annu. Rev. Plant Biol.* 53 449–475. 10.1146/annurev.arplant.53.100301.135233 12221984

[B87] Steglich C. Futschik M. E. Lindell D. Voss B. Chisholm S. W. Hess W. R. (2008). The challenge of regulation in a minimal photoautotroph: non-coding RNAs in *Prochlorococcus*. *PLoS Genet.* 4:e1000173. 10.1371/journal.pgen.1000173 18769676 PMC2518516

[B88] Storz G. (2002). An expanding universe of noncoding RNAs. *Science* 296 1260–1263. 10.1126/science.1072249 12016301

[B89] Storz G. Opdyke J. A. Zhang A. (2004). Controlling mRNA stability and translation with small, noncoding RNAs. *Curr. Opin. Microbiol.* 7 140–144. 10.1016/j.mib.2004.02.015 15063850

[B90] Stougaard P. Molin S. Nordström K. (1981). RNAs involved in copy-number control and incompatibility of plasmid R1. *Proc. Natl. Acad. Sci. U.S.A.* 78 6008–6012. 10.1073/pnas.78.10.6008 6171808 PMC348966

[B91] Sugita C. Ogata K. Shikata M. Jikuya H. Takano J. Furumichi M. (2007). Complete nucleotide sequence of the freshwater unicellular cyanobacterium *Synechococcus elongatus* PCC 6301 chromosome: gene content and organization. *Photosynth. Res.* 93 55–67. 10.1007/s11120-006-9122-4 17211581

[B92] Sun T. Li S. Song X. Pei G. Diao J. Cui J. (2018). Re-direction of carbon flux to key precursor malonyl-CoA via artificial small RNAs in photosynthetic *Synechocystis* sp. PCC 6803. *Biotechnol. Biofuels* 11:26. 10.1186/s13068-018-1032-0 29441124 PMC5798194

[B93] Sun T. Pei G. Wang J. Chen L. Zhang W. (2017). A novel small RNA CoaR regulates coenzyme A biosynthesis and tolerance of *Synechocystis* sp. PCC6803 to 1-butanol possibly via promoter-directed transcriptional silencing. *Biotechnol. Biofuels* 10:42. 10.1186/s13068-017-0727-y 28239414 PMC5319066

[B94] Sun X. Zhulin I. Wartell R. M. (2002). Predicted structure and phyletic distribution of the RNA-binding protein Hfq. *Nucleic Acids Res.* 30 3662–3671. 10.1093/nar/gkf508 12202750 PMC137430

[B95] Tafer H. Hofacker I. L. (2008). RNAplex: a fast tool for RNA-RNA interaction search. *Bioinformatics* 24 2657–2663. 10.1093/bioinformatics/btn193 18434344

[B96] Tamagnini P. Axelsson R. Lindberg P. Oxelfelt F. Wünschiers R. Lindblad P. (2002). Hydrogenases and hydrogen metabolism of cyanobacteria. *Microbiol. Mol. Biol. Rev.* 66 1–20. 10.1128/MMBR.66.1.1-20.2002 11875125 PMC120778

[B97] Tjaden B. (2008). TargetRNA: a tool for predicting targets of small RNA action in bacteria. *Nucleic Acids Res.* 36 W109–W113. 10.1093/nar/gkn264 18477632 PMC2447797

[B98] Tjaden B. Goodwin S. S. Opdyke J. A. Guillier M. Fu D. X. Gottesman S. (2006). Target prediction for small, noncoding RNAs in bacteria. *Nucleic Acids Res.* 34 2791–2802. 10.1093/nar/gkl356 16717284 PMC1464411

[B99] Tomizawa J. Itoh T. Selzer G. Som T. (1981). Inhibition of ColE1 RNA primer formation by a plasmid-specified small RNA. *Proc. Natl. Acad. Sci. U.S.A.* 78 1421–1425. 10.1073/pnas.78.3.1421 6165011 PMC319142

[B100] Tous C. Vega-Palas M. A. Vioque A. (2001). Conditional expression of RNase P in the cyanobacterium *Synechocystis* sp. PCC6803 allows detection of precursor RNAs. *J. Biol. Chem.* 276 29059–29066. 10.1074/jbc.M103418200 11384989

[B101] Tyagi J. S. Kinger A. K. (1992). Identification of the 10Sa RNA structural gene of *Mycobacterium tuberculosis*. *Nucleic Acids Res.* 20 138–138. 10.1093/nar/20.1.138 1371186 PMC310338

[B102] Ushida C. Himeno H. Watanabe T. Muto A. (1994). tRNA-like structures in 10Sa RNAs of *Mycoplasma capricolum* and *Bacillus subtilis*. *Nucleic Acids Res.* 22 3392–3396. 10.1093/nar/22.16.3392 7521527 PMC523734

[B103] Valentin-Hansen P. Eriksen M. Udesen C. (2004). The bacterial Sm-like protein Hfq: a key player in RNA transactions. *Mol. Microbiol.* 51 1525–1533. 10.1111/j.1365-2958.2003.03935.x 15009882

[B104] Volpe T. A. Kidner C. Hall I. M. Teng G. Grewal S. I. Martienssen R. A. (2002). Regulation of heterochromatic silencing and histone H3 lysine-9 methylation by RNAi. *Science* 297 1833–1837. 10.1126/science.1074973 12193640

[B105] Voss B. Georg J. Schon V. Ude S. Hess W. R. (2009). Biocomputational prediction of non-coding RNAs in model cyanobacteria. *BMC Genomics* 10:123. 10.1186/1471-2164-10-123 19309518 PMC2662882

[B106] Voss B. Gierga G. Axmann I. M. Hess W. R. (2007). A motif-based search in bacterial genomes identifies the ortholog of the small RNA Yfr1 in all lineages of cyanobacteria. *BMC Genomics* 8:375. 10.1186/1471-2164-8-375 17941988 PMC2190773

[B107] Wagner E. G. H. Altuvia S. Romby P. (2002). 12 Antisense RNAs in bacteria and their genetic elements. *Adv. Genet.* 46 361–398. 10.1016/S0065-2660(02)46013-0 11931231

[B108] Wang Q. Jantaro S. Lu B. Majeed W. Bailey M. He Q. (2008). The high light-inducible polypeptides stabilize trimeric photosystem I complex under high light conditions in *Synechocystis* PCC 6803. *Plant Physiol.* 147 1239–1250. 10.1104/pp.108.121087 18502976 PMC2442545

[B109] Wassarman K. M. Zhang A. Storz G. (1999). Small RNAs in *Escherichia coli*. *Trends Microbiol.* 7 37–45. 10.1016/S0966-842X(98)01379-1 10068996

[B110] Watanabe T. Sugita M. Sugiura M. (1998). Identification of 10Sa RNA (tmRNA) homologues from the cyanobacterium *Synechococcus* sp. strain PCC6301 and related organisms. *Biochim. Biophys. Acta Biomembranes* 1396 97–104. 10.1016/S0167-4781(97)00180-2 9524235

[B111] Watanabe T. Sugiura M. Sugita M. (1997). A novel small stable RNA, 6Sa RNA, from the cyanobacterium *Synechococcus* sp. strain PCC6301. *FEBS Lett.* 416 302–306. 10.1016/S0014-5793(97)01237-4 9373174

[B112] Waters L. S. Storz G. (2009). Regulatory RNAs in bacteria. *Cell* 136 615–628. 10.1016/j.cell.2009.01.043 19239884 PMC3132550

[B113] Weinberg Z. Barrick J. E. Yao Z. Roth A. Kim J. N. Gore J. (2007). Identification of 22 candidate structured RNAs in bacteria using the CMfinder comparative genomics pipeline. *Nucleic Acids Res.* 35 4809–4819. 10.1093/Nar/Gkm487 17621584 PMC1950547

[B114] Williams K. P. Bartel D. P. (1996). Phylogenetic analysis of tmRNA secondary structure. *RNA* 2 1306–1310. 8972778 PMC1369456

[B115] Wright P. R. Georg J. Mann M. Sorescu D. A. Richter A. S. Lott S. (2014). CopraRNA and IntaRNA: predicting small RNA targets, networks and interaction domains. *Nucleic Acids Res.* 42 W119–W123. 10.1093/nar/gku359 24838564 PMC4086077

[B116] Xu W. Chen H. He C. L. Wang Q. (2014). Deep sequencing-based identification of small regulatory RNAs in *Synechocystis* sp. PCC 6803. *PLoS One* 9:e92711. 10.1371/journal.pone.0092711 24647397 PMC3960264

[B117] Zess E. K. Begemann M. B. Pfleger B. F. (2016). Construction of new synthetic biology tools for the control of gene expression in the cyanobacterium *Synechococcus* sp. strain PCC 7002. *Biotechnol. Bioeng.* 113 424–432. 10.1002/bit.25713 26192329

[B118] Zhan J. Wang Q. (2018). “Photoresponse mechanism in cyanobacteria: key factor in photoautotrophic chassis,” in *Synthetic Biology of Cyanobacteria*, eds Zhang W. Song X. (Singapore: Springer Singapore), 75–96. 10.1007/978-981-13-0854-3_4 30091092

[B119] Zhang P. Allahverdiyeva Y. Eisenhut M. Aro E. M. (2009). Flavodiiron proteins in oxygenic photosynthetic organisms: photoprotection of photosystem II by Flv2 and Flv4 in *Synechocystis* sp. PCC 6803. *PLoS One* 4:e5331. 10.1371/journal.pone.0005331 19390625 PMC2669126

[B120] Zhang P. Eisenhut M. Brandt A. -M. Carmel D. Silén H. M. Vass I. (2012). Operon flv4-flv2 provides cyanobacterial photosystem II with flexibility of electron transfer. *Plant Cell* 24 1952–1971. 10.1105/tpc.111.094417 22570444 PMC3442580

[B121] Zhao Y. Li H. Hou Y. Cha L. Cao Y. Wang L. (2008). Construction of two mathematical models for prediction of bacterial sRNA targets. *Biochem. Biophys. Res. Commun.* 372 346–350. 10.1016/j.bbrc.2008.05.046 18501192

